# Prognostic role of platelet to lymphocyte ratio in prostate cancer: A meta-analysis

**DOI:** 10.1097/MD.0000000000012504

**Published:** 2018-10-05

**Authors:** Jiangfeng Wang, Xiaofeng Zhou, Yuhui He, Xing Chen, Naibo Liu, Zhenshan Ding, Junjie Li

**Affiliations:** Department of Urology, China–Japan Friendship Hospital, Beijing, China.

**Keywords:** meta-analysis, platelet-to-lymphocyte ratio, prognosis, prostate cancer

## Abstract

**Background:**

Recently, the prognostic value of the platelet-to-lymphocyte ratio (PLR) has been identified in multiple cancers. However, the prognostic significance of the PLR in prostate cancer (PCa) remains conflicting. We therefore searched relevant studies and conducted a meta-analysis.

**Methods:**

Papers from the databases of PubMed, Web of Science, and the Cochrane Library were retrieved. Six studies comprising 1324 patients were included.

**Results:**

The pooled analysis demonstrated that an elevated PLR predicted poor overall survival (OS; HR = 1.85, 95% CI = 1.51–2.25, *P < *.001) and disease-free survival (DFS; HR = 1.4, 95% CI = 1.1–1.79, *P = *.007). Subgroup analyses showed that the PLR remained a significant prognostic factor for OS irrespective of ethnicity, tumor stage, or cut-off value. The PLR was an indicator of poor DFS in Asian patients, but not in Caucasian patients. No significant publication bias was detected.

**Conclusion:**

This meta-analysis showed that a high PLR was correlated with poor DFS and OS in patients with prostate cancer. Due to this meta-analysis being derived from a few studies, the results should be validated in clinical practice.

## 1 Introduction

Prostate cancer (PCa) is the second most prevalent cancer and ranks as the fifth cause of cancer-related death in men worldwide.^[1]^ Early detection with PSA screening could identify PCa cases^[2]^ and patients with localized PCa could be treated with active surveillance, radical prostatectomy, or external-beam radiotherapy, as appropriate.^[3]^ However, when the disease becomes metastatic, androgen deprivation therapy (ADT) is usually applied and the resistance to ADT leads to unfavorable prognosis of this disease.^[4]^ A variety of molecular biomarkers are adopted to stratify risk patients and predict survival outcomes.^[5]^ These biological markers need to be detected by special apparatuses and are therefore inconvenient to use in daily practice. Thus, identification of cost-effective and convenient prognostic markers is essential for individualized treatment of PCa.

Inflammation is one of the most important and well-recognized factors leading to cancer development.^[6]^ In recent years, a number of studies have focused on hematological parameters, which can reflect the status of immune responses in cancer patients.^[7–9]^ These indices include plasma fibrinogen, C-reactive protein (CRP) levels, neutrophil-to-lymphocyte ratio (NLR), and platelet-to-lymphocyte ratio (PLR), among which NLR and PLR are widely investigated in various cancers because they are readily available and do not add extra costs.^[9–13]^ The PLR is calculated as the platelet count divided by the lymphocyte count and can be obtained from routine blood tests. Previous studies have explored the prognostic role of PLR in patients with PCa, although the results are controversial.^[14–19]^ To perform an insightful investigation on the association of PLR and clinical outcomes in PCa, we thoroughly searched relevant publications and conducted a quantitative pooled analysis.

## 2 Materials and methods

### 2.1 Literature search

This meta-analysis was carried out under the guideline of the Preferred Reporting Items for Systematic Reviews and Meta-Analyses (PRISMA) statement.^[20]^ A comprehensive literature search was conducted of the PubMed, Web of Science, and Cochrane Library databases. The search strategies were based on the combination of the following items: “platelet-to-lymphocyte ratio,” “platelet-lymphocyte ratio,” “PLR,” “prostatic neoplasms,” “prostate cancer,” “prognosis,” “survival,” and “outcome.” The last search was updated on November 2, 2017. Meanwhile, to identify possible inclusions, reference lists were also manually examined. Ethical approval was waived because we did not make any clinical research in this manuscript, we just collected the data from available publications.

### 2.2 Inclusion and exclusion criteria

The inclusion criteria were as follows: PCa was diagnosed based on pathological methods; a dichotomous cut-off value of the PLR was identified to classify the patients into high and low PLR groups; HR and 95% CI for the PLR in OS and/or DFS were provided or could be calculated from sufficient data;^[21]^ published in English; and (5) blood or serum specimens were used to determine PLR. The exclusion criteria were: meeting abstracts, reviews, letters, or nonclinical studies; studies without sufficient data to estimate HR and 95% CI; or duplicate studies.

### 2.3 Data extraction and quality assessment

All candidate reports were evaluated by 2 independent investigators, and any discrepancies were resolved by consensus. The following information was extracted from each eligible study: first author's name, year of publication, study location, cases of patients, tumor stage, study design, study duration, cut-off value of the PLR, types of survival analysis, and HRs and 95% CIs of OS and/or DFS. Notably, if both HRs and 95% CIs on multivariate (MV) and univariate (UV) analyses were presented, the MV HR and 95% CI were adopted; UV HR and 95% CI were used only if MV values were not provided. The NOS was utilized to assess the quality of eligible studies. The NOS estimates studies based on 3 parts: selection (0–4 stars), comparability (0–stars), and outcome assessment (0–3 stars). The maximum score of NOS is 9 and studies with scores ≥6 are considered as high quality.

### 2.4 Statistical analysis

For this meta-analysis, pooled HR with 95%CI was used as effective measures. Heterogeneity among studies was evaluated by using Cochran's *Q* test^[22]^ and Higgins *I*-squared statistic.^[23]^ A *P*_heterogeneity_ < .10 or *I*^2^ > 50% suggested significant heterogeneity, and a random-effect model was used to pool HRs and 95%CIs. Otherwise, a fixed-effect model was selected. Subgroup analysis stratified by ethnicity, tumor stage, and PLR cut-off value was also conducted for detailed information. Publication bias was determined using Begg's funnel plot^[24]^ and Egger's linear regression test.^[25]^ All statistical analyses were performed by using Stata version 12 (STATA, College Station, TX) and *P < *.05 was considered as statistically significant.

## 3 Results

### 3.1 Included studies and study characteristics

**Figure 1:**
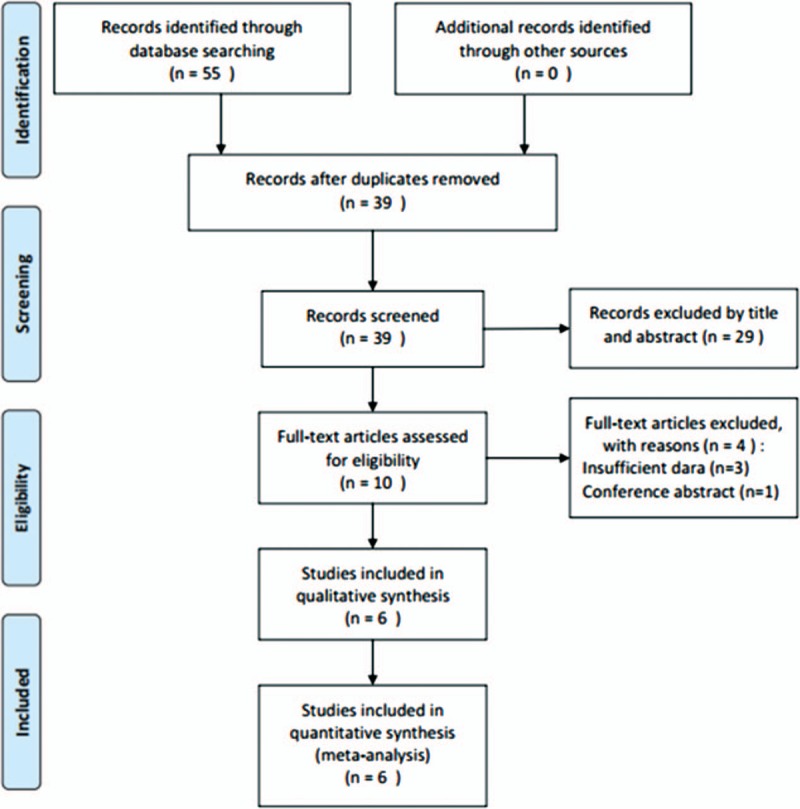
Flow diagram showing the selection of literature for the meta-analysis.

A flowchart of the literature selection process is shown in Figure 1. The initial search of electronic databases identified 55 records; after duplicates were removed, 39 papers remained. Twenty-nine papers were then excluded by screening titles and/or abstracts. Ten full-text articles were further examined for eligibility and 4 articles were excluded because 3 lacked survival data and one was a meeting abstract. At last, 6 articles published between 2015 and 2017 were included for meta-analysis. Three studies were performed in China,^[14,16,18]^ one was conducted in Spain,^[15]^ one in Italy,^[17]^ and one in Austria.^[19]^ All 6 studies reported data on overall survival (OS) and 4 studies showed data on disease-free survival (DFS).^[14–16,19]^ The cut-off values of PLR ranged from 117.58 to 210. All studies had a retrospective study design and a Newcastle–Ottawa scale (NOS) score ≥6. The baseline characteristics of the included studies are shown in Table 1.

**Table 1:**
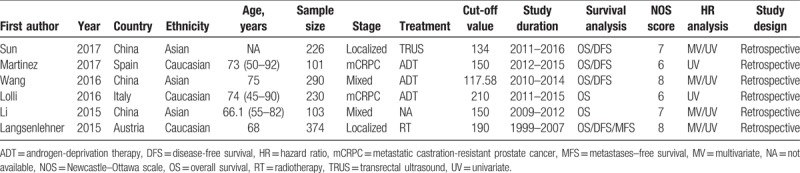
Study characteristics.

### 3.2 Prognostic effect of PLR on OS

**Figure 2:**
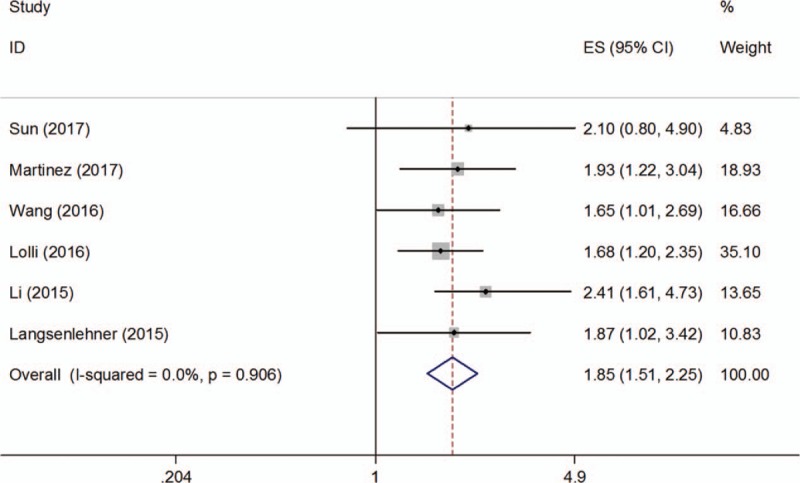
Forest plot reflecting the association between PLR and OS. OS = overall survival, PLR = platelet-to-lymphocyte ratio.

Six studies^[14–19]^ with a total of 1324 patients investigated the association of PLR and OS in PCa. Because of the lack of heterogeneity (*I*^2^ = 0, *P = *.906), a fixed-effects model was used. As shown in Figure 2, the combined hazard ratio (HR) was 1.85, with 95% confidence interval (CI) = 1.51–2.25, *P < *.001. Subgroup analyses showed that PLR remained a significant prognostic factor for OS irrespective of ethnicity, tumor stage, and cut-off value (Table 2).

**Table 2:**
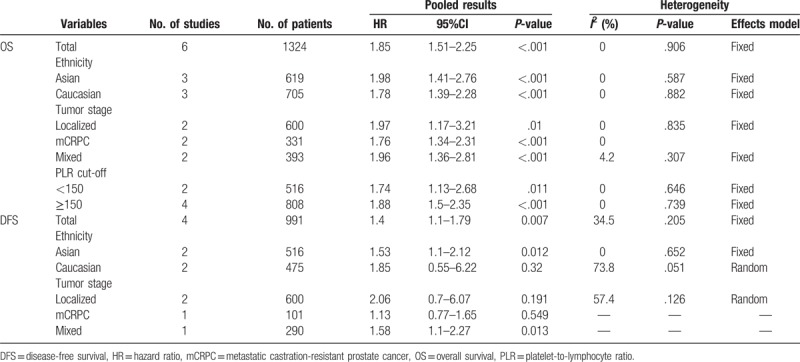
The pooled results of subgroups for the association between PLR and OS and DFS in prostate cancer.

### 3.3 Prognostic impact of PLR on DFS

**Figure 3:**
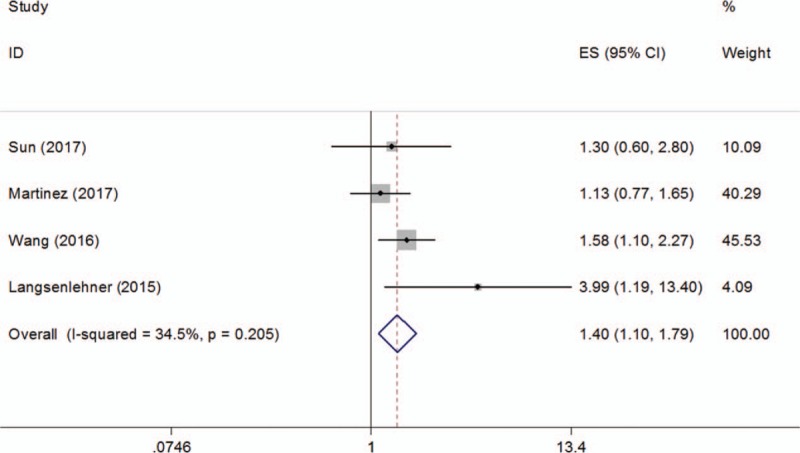
Forest plot reflecting the association between PLR and DFS. DFS = disease-free survival, PLR = platelet-to-lymphocyte ratio.

There were 4 studies^[14–16,19]^ comprising 991 patients analyzing the prognostic significance of PLR on DFS. No significant heterogeneity (*I*^2^ = 34.5%, *P = *.205) was detected and a fixed-effects model was utilized. The combined results were: HR = 1.4, 95% CI = 1.1–1.79, *P = *.007 (Fig. 3, Table 2). The results of subgroup analysis suggested that a high PLR was an indicator of poor DFS in Asian patients (HR = 1.53, 95% CI = 1.1–2.12, *P = *.012), but not in Caucasian patients (HR = 1.85, 95% CI = 0.55–6.22, *P = *.32) (Table 2). The subgroup analysis on tumor stage showed that PLR was a prognostic marker for mixed stage patients (HR = 1.58, 95% CI = 1.1–2.27, *P = *.013) (Table 2), because only one study was included for this analysis, the results should be further verified.

### 3.4 Publication bias

**Figure 4:**
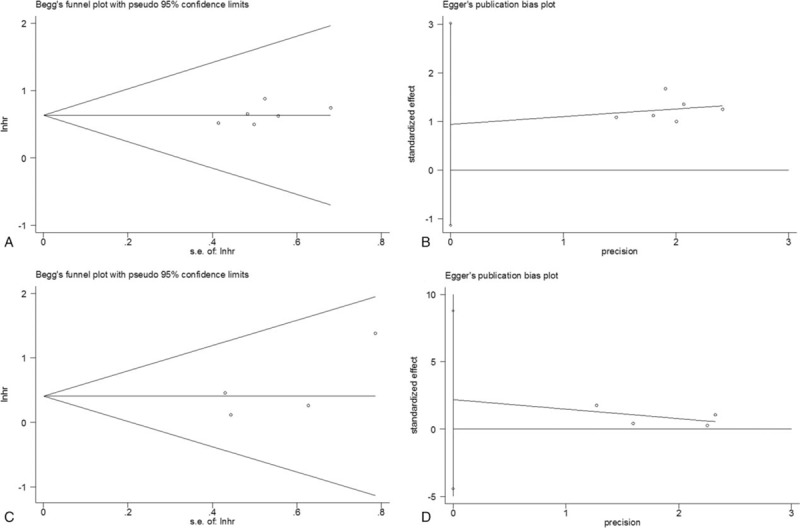
Funnel plot and linear regression test for publication bias. (A) Begg's funnel plot for OS; (B) Egger's linear regression test for OS; (C) Begg's funnel plot for DFS; and (D) Egger's linear regression test for DFS. DFS = disease-free survival, OS = overall survival.

Both Begg's funnel plot and Egger's linear regression test were conducted to estimate publication bias. As shown in Figure 4, no publication bias was detected for OS (Begg's *P = *.26, Egger's *P = *.276) or for DFS (Begg's *P = *.734, Egger's *P = *.291). The data suggested that there was no significant publication bias in our study.

## 4 Discussion

Because of the controversial results on PLR and survival analysis of PCa from previous studies, we performed a meta-analysis. The pooled results demonstrated that elevated PLR was associated with poor OS and DFS. In addition, PLR showed efficient prognostic value for OS, whereas it predicted poor DFS for Asian patients. To the best of our knowledge, this is the first meta-analysis to investigate the prognostic significance of PLR in patients with prostate cancer.

Underlying infections and inflammatory responses are evaluated to be correlated with 15% to 20% of all cancer-related deaths worldwide.^[26,27]^ Chronic inflammation increases the risk of developing cancer and also promotes cancer progression and metastasis.^[28,29]^ The changes of peripheral blood counts can reflect the inflammatory responses in cancer patients. Recent studies suggested that platelets can mediate cancer cell growth, dissemination, and angiogenesis.^[30]^ Direct interactions of platelets and tumor cells can facilitate tumor metastasis. Platelet-derived transforming growth factor (TGF)-β and the contacts between platelets and cancer cells synergistically activate the TGFβ/Smad and nuclear factor (NF)-κB pathways in cancer cells, leading to the enhancement of metastatic capability of cancer cells.^[31]^ Furthermore, aggregation of platelets around tumor cells could protect tumor cells from lysis by natural killer (NK) cells.^[32]^ On the contrary, tumor-infiltrating cluster of differentiation (CD) 8+ and CD4+ lymphocytes are proven to exert important effects on antitumor activity.^[33]^ In addition, an increased number of tumor-infiltrating lymphocytes (TILs) is associated with better prognosis in various cancers including breast cancer and colorectal cancer.^[7,34]^ As a parameter that combines platelet counts and lymphocyte counts, the PLR could provide relatively accurate information on the prognosis of patients with cancer. In addition, this index is readily available in daily practice.

Hematological indices such as the NLR and PLR have attracted much attention due to their high cost-effectiveness for prognostications in cancer.^[9,11]^ As for PCa, a previous meta-analysis has demonstrated that an increased NLR predicts poor OS and progression-free survival (PFS)/recurrence-free survival (RFS) in PCa.^[35]^ However, the prognostic role of the PLR in PCa remains controversial. Langsenlehner et al^[19]^ reported that an increased PLR acts as an indicator for poor OS in patients with PCa treated with radiotherapy. Additionally, Wang et al. also showed that the PLR is an independent prognostic factor for PFS and OS.^[16]^ However, Sun's work indicated a nonsignificant correlation of the PLR with DFS and OS in PCa.^[14]^ In the current meta-analysis, we collected the conflicting data and synthesized them using quantitative methods. Our results confirmed the significant prognostic role of the PLR for DFS and OS. Notably, a series of studies also investigated the association between the PLR and survival in various cancers through meta-analysis. For example, Zhu et al^[36]^ showed that the PLR could serve as an indicator of poor prognosis in patients with breast cancer. Huang et al^[37]^ revealed that an elevated PLR predicted poor prognosis and clinicopathological characteristics in colorectal cancer. The results of this meta-analysis were in line with previous studies, suggesting that the PLR should be considered as an applicable prognostic factor in diverse solid tumors. In addition, regarding the prognostic role of PLR in DFS, on tumor stage, 2 studies included localized stage patients and one included mCRPC patients and one included mixed patients. Because only one study was on mCRPC and mixed patients, respectively, therefore, the subgroup analysis could be less convincing by tumor stage. In the ethnicity subgroup, PLR showed prognostic value for DFS on Asian patients, but not on Caucasian patients. Although one Asian study included localized patients and the other included mixed patients. The detailed analysis could not be performed because of limited data from included studies.

There were several limitations in the present study. First, the number of included studies was relatively small. Although we conducted a comprehensive search of the literature, only 6 studies were included. Second, all eligible studies were retrospective studies, and no randomized controlled trials (RCTs) were included. Therefore, the quality of eligible studies is a concern. Third, different cut-off values of the PLR were used in the included studies, which might contribute to the heterogeneity for this meta-analysis. Fourth, 4 out of all 6 included studies showed positive results, although no publication bias was detected using Begg's and Egger's tests. More eligible studies with conflicting results are needed to validate the results of the present study.

In conclusion, a high PLR was correlated with poor DFS and OS in patients with prostate cancer. Considering that the PLR is a convenient and low-cost hematological marker, we recommend that the PLR could be applied as a factor to provide reliable information about patients with PCa.

## Author contributions

**Conceptualization:** Xiaofeng Zhou.

**Data curation:** Jiangfeng Wang, Xiaofeng Zhou, Xing Chen, Zhenshan Ding.

**Formal analysis:** Jiangfeng Wang, Xiaofeng Zhou, Xing Chen, Naibo Liu, Zhenshan Ding.

**Funding acquisition:** Yuhui He, Xing Chen, Naibo Liu, Zhenshan Ding, Junjie Li.

**Investigation:** Jiangfeng Wang, Yuhui He, Junjie Li.

**Methodology:** Xiaofeng Zhou, Xing Chen, Naibo Liu, Junjie Li.

**Project administration:** Zhenshan Ding.

**Resources:** Xing Chen, Naibo Liu.

**Software:** Jiangfeng Wang, Yuhui He, Naibo Liu, Junjie Li.

**Supervision:** Jiangfeng Wang, Yuhui He, Zhenshan Ding, Junjie Li.

**Validation:** Xing Chen, Zhenshan Ding, Junjie Li.

**Visualization:** Yuhui He, Xing Chen, Naibo Liu, Junjie Li.

**Writing – original draft:** Yuhui He, Naibo Liu.

**Writing – review & editing:** Zhenshan Ding, Junjie Li.
